# Constructing lncRNA functional similarity network based on lncRNA-disease associations and disease semantic similarity

**DOI:** 10.1038/srep11338

**Published:** 2015-06-10

**Authors:** Xing Chen, Chenggang Clarence Yan, Cai Luo, Wen Ji, Yongdong Zhang, Qionghai Dai

**Affiliations:** 1 National Center for Mathematics and Interdisciplinary Sciences, Chinese Academy of Sciences, Beijing, 100190, China; 2 Academy of Mathematics and Systems Science, Chinese Academy of Sciences, Beijing, 100190, China; 3 Department of Automation, Tsinghua University, Beijing, 100084, China; 4 Institute of Computing Technology, Chinese Academy of Sciences, Beijing, 100190, China; 5 Key Lab of Intelligent Information Processing of Chinese Academy of Sciences, Institute of Computing Technology, Chinese Academy of Sciences, Beijing, 100190, China

## Abstract

Increasing evidence has indicated that plenty of lncRNAs play important roles in many critical biological processes. Developing powerful computational models to construct lncRNA functional similarity network based on heterogeneous biological datasets is one of the most important and popular topics in the fields of both lncRNAs and complex diseases. Functional similarity network consturction could benefit the model development for both lncRNA function inference and lncRNA-disease association identification. However, little effort has been attempted to analysis and calculate lncRNA functional similarity on a large scale. In this study, based on the assumption that functionally similar lncRNAs tend to be associated with similar diseases, we developed two novel lncRNA functional similarity calculation models (LNCSIM). LNCSIM was evaluated by introducing similarity scores into the model of Laplacian Regularized Least Squares for LncRNA–Disease Association (LRLSLDA) for lncRNA-disease association prediction. As a result, new predictive models improved the performance of LRLSLDA in the leave-one-out cross validation of various known lncRNA-disease associations datasets. Furthermore, some of the predictive results for colorectal cancer and lung cancer were verified by independent biological experimental studies. It is anticipated that LNCSIM could be a useful and important biological tool for human disease diagnosis, treatment, and prevention.

There are estimated 20,000 protein-coding genes in the human genome, which account for only approximately 1.5% of the whole genome^1^,^2^,^3^,^4^,^5^,^6^,^7^,^8^,^9^. Therefore, more than 98% of the human genome does not encode protein sequences. Furthermore, plenty of evidences have demonstrated the critical regulative roles of noncoding RNAs (ncRNAs) in a broad range of fundamental and important biological processes^10^, which challenge the traditional view that RNA is just transcriptional noise and intermediary between gene and protein^11^,^12^. Especially, Taft *et al.* observed that the proportion of non-protein-coding sequence correspondingly increases with increased complexity of organisms^13^. Based on transcript lengths, ncRNAs can be divided into small ncRNAs and long ncRNAs. Long noncoding RNAs (lncRNAs) are defined as a class of important heterogeneous ncRNAs with the length more than 200 nucleotides^6^,^14^,^15^,^16^,^17^,^18^,^19^, which make up the largest fraction of the mammalian noncoding transcriptome^10^,^14^. Based on traditional gene mapping approaches, H19 and Xist were discovered in the early 1990 s^20^,^21^,^22^,^23^. However, these two lncRNAs were considered to be rare exceptions to the central dogma of molecular biology at that time. Guttman *et al.* used chromatin-state maps to develop a new genome wide approach for lncRNAs discovery and identified 1,600 large intervening non-coding RNAs (lincRNAs) across four mouse cell types^24^. Furthermore, a functional genomic approach has been developed to assign putative functions to each lincRNA, showing these lincRNAs has played various roles in fundamental and important biological processes^24^. Based on chromatin marks and RNA-sequencing (RNA-seq) data, Cabili *et al.* presented an integrative approach to generate the human lincRNA catalog, which included more than 8000 lincRNAs across 24 different human cell types and tissues^25^. These lincRNAs have also been characterized by a panorama of more than 30 properties, such as sequence, structural, transcriptional, and orthology features^25^. Nowadays, a lot of lncRNAs have been identified in eukaryotic organisms ranging from nematodes to humans, which benefits from the rapid development of both experimental technology and computational algorithms^18^,^24^,^26^,^27^,^28^,^29^. In comparison with protein-coding genes, lncRNAs tend to show a relatively lower expression level but much more tissue-specific pattern^12^,^16^,^27^,^30^,^31^,^32^,^33^,^34^,^35^,^36^. Furthermore, lncRNAs tend to be less conserved across species and have longer, but fewer, exons^18^,^23^,^25^.

Because of the low cross-species conservation, low expression levels and high tissue specificity of lncRNAs, people often argued against the functionality of lncRNAs in the past^12^,^37^. Increasing number of experimental studies in recent years have shown that plenty of lncRNAs are not transcriptional noise but play important roles in many critical biological processes, including transcriptional and post-transcriptional regulation, epigenetic regulation, organ or tissue development, cell differentiation, cell cycle control, cellular transport, metabolic processes, chromosome dynamics and so on^7^,^8^,^9^,^11^,^14^,^15^,^24^,^29^,^38^,^39^,^40^,^41^,^42^,^43^,^44^,^45^,^46^. Compared with the huge number of lncRNAs annotated by GENCODE^16^,^18^, only a few lncRNAs have been extensively studied, which have shed light on their possible functions and the underlying molecular mechanism of their functions^23^,^46^. Elucidating the functions of lncRNAs is a big challenge for both experimental studies and computational biology^23^. Considering the important roles of lncRNAs in various biological processes, it is no surprise that mutations and dysregulations of lncRNAs have been linked to the development and progression of a broad range of complex human diseases^8^,^12^,^14^,^15^,^47^,^48^,^49^,^50^, such as breast cancer^51^,^52^,^53^,^54^, hepatocellular cancer^55^,^56^,^57^,^58^,^59^,^60^, prostate cancer^61^,^62^,^63^,^64^,^65^, colon cancer^66^, bladder cancer^67^, thyroid cancer^68^, lung cancer^69^,^70^, ovarian cancer^54^, leukemia^71^,^72^, Alzheimer’s diseases^73^, diabetes^74^,^75^, and HIV^76^. lncRNA PCA3 has about 60 times expression levels in prostate tumors compared with normal tissues, therefore PCA3 has been treated as a well-known example of potential cancer diagnostic biomarker^38^,^47^,^64^. Another well-known example is HOTAIR, which is overexpressed from hundreds to nearly two-thousand-fold in breast cancer metastases based on quantitative PCR^53^. Furthermore, HOTAIR is also an independent prognostic marker of hepatocellular cancer recurrence for the patients after liver transplantation^59^. lncRNAs can be used as both potential biomarkers in disease diagnosis, treatment, prognosis and potential drug targets in drug discovery and clinical treatment^47^. So far, although plenty of biological datasets about lncRNA sequence and expression have been generated and stored in some publicly available databases, such as NRED^77^, lncRNAdb^28^, NONCODE^29^, the number of lncRNAs reported to be associated with diseases is still very limited.

Calculating lncRNA functional similarity could benefit the construction of computational model for lncRNA function inference and lncRNA-disease association identification based on the assumption that similar lncRNAs have similar functions and relevance with similar diseases^78^. In this way, potential lncRNA functions and lncRNA-disease associations could be verified based on further experimental validation. Therefore, the time and cost of biological experiments could be significantly reduced. Furthermore, it is well known that lncRNA function inference and disease-lncRNA association identification could benefit lncRNA functions understanding, biomarker identification and drug discovery for human disease diagnosis, treatment, prognosis and prevention. Computational methods have played important roles in ncRNA investigation in plenty of previous successful studies^79^,^80^,^81^,^82^,^83^,^84^,^85^,^86^,^87^,^88^. Therefore, developing powerful computational models based on heterogeneous biological datasets for lncRNA functional similarity calculation and functional network construction is one of the most important and popular topics in the fields of both lncRNAs and complex diseases.

In our previous work, we calculated the lncRNA functional similarity by integrating lncRNA expression similarity based on the Spearman correlation coefficient between the expression profiles of each lncRNA pair and lncRNA Gaussian interaction profile kernel similarity based on the assumption that similar lncRNAs tend to show a similar interaction and non-interaction pattern with the diseases^78^. Based on calculated lncRNA similarity, we further developed Laplacian Regularized Least Squares for LncRNA–Disease Association (LRLSLDA) in the semi-supervised learning framework^78^.

It is well known that genes with similar functions tend to be associated with similar diseases and vice versa^89^. In the recent researches about non-coding RNAs, similar conclusions have been obtained^11^,^78^,^82^,^90^. Based on the logical extension of the basic assumption in the previous disease genes identification, Chen *et al.* and Lu *et al.* proposed and validated the following basic assumption for disease-related lncRNAs and miRNAs prediction: similar diseases tend to be associated with functionally similar lncRNAs and miRNAs and vice versa^78^,^90^, respectively. Therefore, the conclusion can be obtained that the functional similarity between two lncRNAs can be calculated by quantitatively measuring the similarity of diseases associated with these two lncRNAs. In this article, we developed two novel lncRNA functional similarity calculation models (LNCSIM) based on above conclusion. LNCSIM consists of the following two steps. Firstly, we developed two methods to calculate the semantic similarity between different diseases based on the structure of directed acyclic graph (DAG) which represents the relationships among different diseases. Secondly, the functional similarity of two lncRNAs was calculated by measuring the semantic similarity of their associated two groups of diseases. To validate the performance of LNCSIM, we introduced lncRNA functional similarity into the model of LRLSLDA for lncRNA-disease associations prediction developed in the previous work^78^. As a result, the reliable AUCs of 0.8130 and 0.8198 are obtained in the leave-one-out cross validation (LOOCV) of known experimentally confirmed lncRNA-disease association in the LncRNADisease for two versions of lncRNA similarity scores, increasing AUCs of 0.037 and 0.0438 than previous LRLSLDA, respectively. We also applied LRLSLDA with lncRNA functional similarity (LRLSLDAS) to Colorectal cancer and Lung cancer and further implemented global prediction for all the diseases simultaneously. Some of potential lncRNA-disease associations have been confirmed by recent biological experiments. Specially, 80% and 66.67% of top 15 potential associations based on global prediction have been confirmed, respectively, demonstrating the potential value of LNCSIM for disease-related lncRNA prediction and biomarker detection for human disease diagnosis, treatment, prognosis and prevention. Furthermore, when we applied LNCSIM to another lncRNA-disease association dataset in MNDR and integrated dataset consisting of lncRNA-disease associations obtained from LncRNADisease database and MNDR, significant performance improvement has also been demonstrated in the framework of LOOCV.

## Results

### lncRNA functional similarity

**Figure 1:**
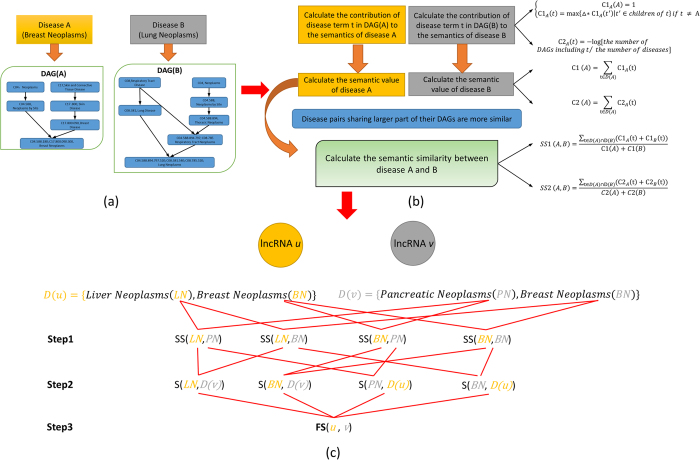
Flowchart of LNCSIM, demonstrating the basic ideas of calculating functional similarity between two lncRNAs. The following three steps have been included: (**a**) identified diseases associated with lncRNA *u* and *v* and constructed their disease DAGs; (**b**) calculated semantic similarity between diseases in the disease groups associated with lncRNA *u* and *v*; (**c**) obtained functional similarity between lncRNA *u* and *v* by calculating the similarity between corresponding disease group associated with each lncRNA. DAG: directed acyclic graph; LN: Liver Neoplasms; BN: Breast Neoplasms; PN: Pancreatic Neoplasms; *D(u)* and *D(v)*: the disease groups associated with lncRNA *u* and *v*; *SS(LN,PN)*: disease semantic similarity matrix between disease LN and PN; *S(LN, D(v))*: the similarity between LN and the disease groups associated with lncRNA *v*: *FS(u,v):* the functional similarity between lncRNA *u* and *v.*

**Figure 2:**
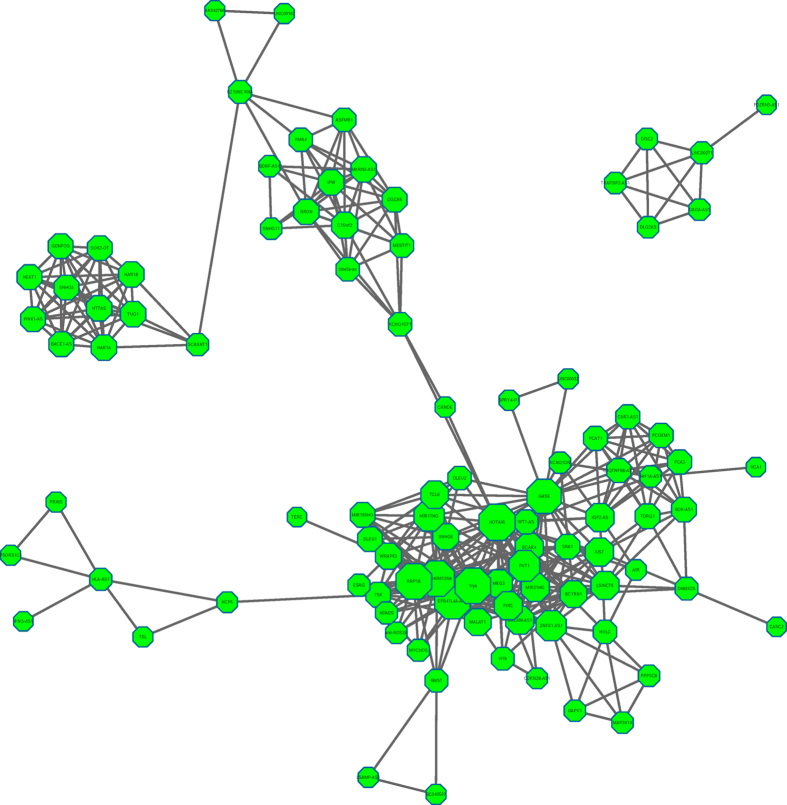
lncRNA functional network was constructed by the model of LNCSIM based on disease semantic similarity model 1, where each node represents one lncRNA and the links was connected if lncRNA pair has a functional similarity equal to or greater than the similarity cutoff (here the cutoff is 0.3 considering the fact that known lncRNA-disease associations is seriously incomplete currently). The size of a node is proportional to the degree of the node. The network is visualized by cytoscape (http://cytoscape.github.io/).

LNSCIM was applied to all the lncRNAs investigated in LncRNADisease database (See Fig. 1). Considering the fact that lncRNA functional similarity was calculated by measuring the semantic similarity of their associated disease groups in the current version of LNCSIM (See Fig. 1 and Methods section), LNCSIM can’t be applied to those lncRNAs without any known associated diseases. Therefore, we selected those lncRNAs with associated diseases in our dataset to implement LNCSIM. Therefore, we obtained the pairwise functional similarity among 104 lncRNAs (see Supplementary Table 1 and 2, respectively). Furthermore, the lncRNA functional network was constructed by setting up a functional similarity threshold and connecting lncRNA pairs with functional similarity greater than or equal to the threshold in the lncRNA functional network (see Fig. 2 and Supplementary Figure 1, respectively).

### Performance evaluation

The effectiveness of LNCSIM was validated by applying the functional similarity results into lncRNA-disease associations prediction based on the model of LRLSLDA developed in our previous work^78^. The aim is to confirm whether the performance of LRLSLDA can be further improved by introducing the information of functional similarity. In the previous version of LRLSLDA, disease similarity and lncRNA similarity scores were derived from Gaussian interaction profile kernel similarity and lncRNA expression similarity. Here, we combined new disease similarity from LNCSIM and disease Gaussian interaction profile kernel similarity into the integrated similarity by a simple mean operation. Furthermore, integrated lncRNA similarity is calculated based on the average of new lncRNA similarity from LNCSIM, lncRNA Gaussian interaction profile kernel similarity, and lncRNA expression similarity. New LRLSLDA models based on two different LNCSIM models were named LRLSLDA-LNCSIM1 and LRLSLDA-LNCSIM2, respectively.

**Figure 3:**
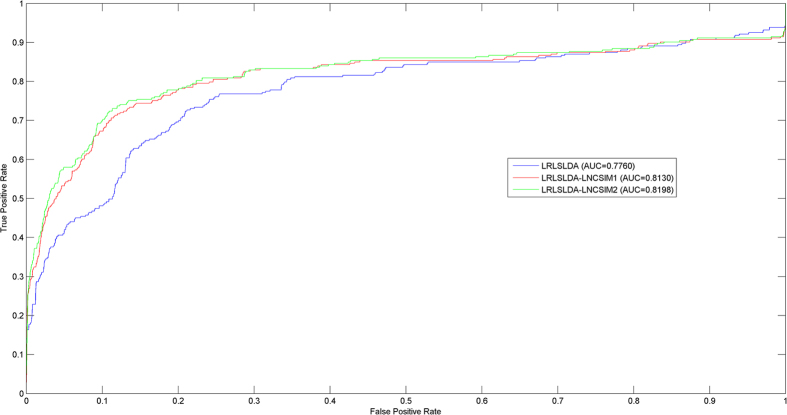
Comparison between LRLSLDA, LRLSLDA-LNCSIM1, and LRLSLDA-LNCSIM2 in terms of ROC curve and AUC based on LOOCV. As a result, new prediction method increase an AUC of 0.037 and 0.0438, respectively, demonstrating that predictive accuracy has been improved by the operation of introducing new disease similarity and lncRNA functional similarity calculated from LNCSIM.

LOOCV was implemented on the known experimentally verified lncRNA-disease associations in the LncRNADisease database to compare the performance of LRLSLDA, LRLSLDA-LNCSIM1, and LRLSLDA-LNCSIM2. As a result, LRLSLDA, LRLSLDA-LNCSIM1, and LRLSLDA-LNCSIM2 achieved AUCs of 0.7760, 0.8130, and 0.8198, respectively (see Fig. 3). New predictive methods increased AUCs of 0.037 and 0.0438, respectively. Therefore, we can reach the conclusion that predictive accuracy has been improved by introducing new disease similarity and lncRNA functional similarity calculated from LNCSIM. In spite of less than two related lncRNAs for each disease on average in the known golden standard dataset, excellent predictive ability of LRLSLDA-LNCSIM1 and LRLSLDA-LNCSIM2 have been demonstrated.

According to Fig. 2, LRLSLDA-LNCSIM1 and LRLSLDA-LNCSIM2 showed similar predictive accuracy. Therefore, we wanted to know whether the similarity results based on LNCSIM1 and LNCSIM2 are complementary. Here, we used the mean, maximum, and minimum of the functional similarity calculated based on LNCSIM1 and LNCSIM2 as integrated functional similarity, respectively. Integrated functional similarity was introduced into the model of LRLSLDA to see whether the predictive performance could be further improved. New LRLSLDA models based on these three kinds of integrated similarity were named LRLSLDA-LNCSIM-mean, LRLSLDA-LNCSIM-max, and LRLSLDA-LNCSIM-min, respectively. We further implemented LOOCV on the known experimentally verified lncRNA-disease associations. As a result, LRLSLDA-LNCSIM-mean, LRLSLDA-LNCSIM-max, and LRLSLDA-LNCSIM-min achieved AUCs of 0.8168, 0.8199, and 0.8132, respectively (see Supplementary Figure 2). No significant performance differences from LRLSLDA-LNCSIM1 and LRLSLDA-LNCSIM2 could be observed, which indicated the similarity results based on LNCSIM1 and LNCSIM2 are not complementary.

### Case studies

We regarded all the known experimentally confirmed lncRNA-disease associations in the LncRNADisease database as training samples and applied LRLSLDA-LNCSIM1 and LRLSLDA-LNCSIM2 to predict potential lncRNAs associated with several important diseases. Furthermore, we tried to search for recent experimental literatures to confirm the predictive results and evaluate the predictive ability of our models.

As the third most common cancer in males and the second in females, colorectal cancer accounts for approximately 8% of all cancer death^91^,^92^,^93^. Colorectal cancer most commonly occurs sporadically and only 25% of the patients have a family disease history, which indicates that lifestyle and environment risk factors could also promote the progression of colorectal cancer^91^,^92^. With the development of high-throughput sequencing technologies in the recent years, researchers have confirmed some critical mutations underlying the pathogenic mechanism of colorectal cancer, including some well-known frequently-mutated oncogenes or tumor suppressor genes (such as APC, KPRS, PIK3CA, and TP53) and a large number of mutated genes with a low frequency^94^,^95^,^96^. Nowadays, biological experiments have further linked mutations and dysregulations of some lncRNAs with the development and progression of colorectal cancer, such as HOTAIR, KCNQ1OT1, and MALAT1 in our training samples. For example, several independent experiments showed that HOTAIR could be considered as a negative prognostic marker in the blood of colorectal cancer patients^23^,^97^,^98^,^99^,^100^. We implemented LRLSLDA-LNCSIM1 and LRLSLDA-LNCSIM2 to prioritize candidate lncRNAs without the known relevance to colorectal cancer. As a result, four out of top 10 predicted colorectal cancer-related lncRNAs (CRNDE, H19, PVT1, and CASC2) have been confirmed to be associated with colorectal cancer based on recent experimental literatures^101^,^102^,^103^ (http://cpfd.cnki.com.cn/Article/CPFDTOTAL-KAXH201309001039.htm). For example, elevated expression of CRNDE in the tissue and plasma of almost all colorectal adenomas and adenocarcinomas has been detected based on microarray analysis, which showed CRNDE has the potential to be a biomarker for colorectal adenomas and cancers^101^. Furthermore, real time PCR demonstrated PVT1 may be a new oncogene and has the functional correlation with the proliferation and apoptosis of colorectal cancer cells^102^.

As the most common cause of cancer-related death worldwide in both men and women, there are estimated 1.4 million deaths resulting from lung cancer each year^104^,^105^,^106^,^107^. Lung cancer death is greater than the combination of following three most cancers: colon, breast, and prostate cancer^104^. Specially, five-year survival rate of lung cancer patients is only approximately 15% from the time of diagnosis, which is lower than other cancers types^104^,^105^,^108^,^109^. Furthermore, considering the important fact that lung cancer patients are not usually diagnosed until advanced stage and there are only few effective lung cancer risk biomarkers, it is necessary and urgent to investigate the mechanism of lung cancer and find new biomarkers for early diagnoses^104^,^105^,^110^,^111^,^112^. In the last decades, much attention has been paid to identify deregulation of protein-coding genes as diagnostic and therapeutic targets of lung cancer^113^. However, with the rapid development of lncRNA discovery and lncRNA function annotation, researchers have found that lncRNA plays a critical role in the development and progression of lung cancer^49^,^114^. Four known lung cancer related lncRNAs has been included in the golden standard dataset. For example, it has been observed that lncRNA BCYRN1 was expressed in the tissues of the breast, cervix, oesophagus, lung, ovary, parotid, and tongue cancer, respectively^115^. However, BCYRN1 was expressed not in corresponding normal tissues^115^. Another example is the association between lncRNA H19 and lung cancer. Based on a knockdown approach, experiments indicated that breast and lung cancer cell clonogenicity and anchorage-independent growth were decreased because of the down-regulation of H19^51^. We further prioritized candidate lncRNAs based on the scored calculated based on LRLSLDA-LNCSIM1 and LRLSLDA-LNCSIM2. Three out of top ten predicted lung cancer related lncRNAs (HOTAIR, UCA1, and GAS5) have been confirmed by independent experimental literatures^102^,^116^,^117^,^118^. A typical example is HOTAIR, which is ranked 2nd by both models. The expression of HOTAIR was upregulated in lung cancer cells based on a three-dimensional organotypic culture model^116^,^118^. One important fact must be pointed out that the known lncRNA-disease association dataset used in this paper for potential association prediction was generated before the publication of this paper. Therefore, this example could be considered as an independent validation of our model. Another biological experiment implemented in 72 NSCLC specimens by qRT-PCR revealed the expression of the tumor suppressor lncRNA GAS5 was significantly down-regulated in lung cancer tissues compared to adjacent noncancerous tissues^117^. Therefore, GAS5 is considered to be a potential diagnostic biomarker for lung cancer and a novel therapeutic target in patients with lung cancer^117^.

As a global ranking method, LRLSLDA-LNCSIM1 and LRLSLDA-LNCSIM2 can reconstruct the missing lncRNA-disease associations for all the diseases simultaneously. Therefore, these two models were applied to simultaneously rank all the candidate lncRNA-disease associations. Out of top 15 potential lncRNA-disease associations, 12 and 10 associations predicted by LRLSLDA-LNCSIM1 and LRLSLDA-LNCSIM2 have been confirmed by experimental literature, respectively (see Table 1 and Supplementary Table 3). Potential association between lncRNA MEG3 and heroin abuse was ranked 2nd and 4th out of 19413 candidate lncRNA-disease pairs by LRLSLDA-LNCSIM1 and LRLSLDA-LNCSIM2, respectively. Quantitative PCR confirmed our predictive result by demonstrating MEG3 was upregulated in human heroin abusers compared to matched drug-free control subjects^119^. Similar high-ranking evidences can also be found in our predictive list, which demonstrate the reliable performance by integrating LNCSIM and LRLSLDA.

We have demonstrated reliable performance of LRLSLDA-LNCSIM1 and LRLSLDA-LNCSIM2 in the terms of LOOCV and the case studies of colorectal cancer and lung cancer. Therefore, we further implemented these two models to prioritize all the candidate lncRNAs for all the diseases in the LncRNADisease database by using all the known experimentally confirmed lncRNA-disease associations in the LncRNADisease database as training samples. Potential human disease-lncRNA association list for each disease were publicly released to benefit the biological experimental validation (see Supplementary Table 4 and 5). It is anticipated that potential disease-lncRNA associations predicted by our models could be confirmed by biological experiments and useful for complex disease research.

### Further performance evaluation on another dataset

To further analysis and validate the results of LNCSIM, we applied LNCSIM to all the lncRNAs investigated in the manually curated diverse ncRNA-disease repository (MNDR)^120^. Pairwise functional similarity among 95 lncRNAs calculated based on two versions of LNCSIM was listed in Supplementary Table 6 and 7, respectively. Furthermore, we integrated the dataset in the LncRNADisease database and MNDR and implement LNCSIM to calculate lncRNA functional similarity among 169 lncRNAs investigated in the integrated dataset (see Supplementary Table 8 and 9, respectively).

Furthermore, LOOCV was implemented on the known experimentally verified lncRNA-disease associations in MNDR to compare the performance of LRLSLDA, LRLSLDA-LNCSIM1, and LRLSLDA-LNCSIM2 (see Supplementary Figure 3). Also for the integrated dataset, LOOCV was implemented based on LRLSLDA, LRLSLDA-LNCSIM1, and LRLSLDA-LNCSIM2 (see Supplementary Figure 4). It could be easily concluded that predictive accuracy has been improved by new disease similarity and lncRNA functional similarity calculated from LNCSIM.

## Discussions

Quantitatively calculating lncRNA functional similarity is critical for lncRNA functions prediction and potential lncRNA-disease associations inference. Therefore, it has become an important goal and significant problem for computational biology research. In this article, the model of LNCSIM was developed to calculate lncRNA functional similarity on a large scale by integrating known lncRNA-disease associations and disease semantic similarity. LNCSIM was motivated based on the basic assumption that functionally similar lncRNAs tend to be associated with similar diseases and hence the lncRNA functional similarity can be calculated by measuring the similarity of diseases associated with them^78^,^90^. Furthermore, LNCSIM was introduced into lncRNA-disease association identification model LRLSLDA developed in our previous work to check whether the predictive performance of LRLSLDA can be further improved. The reliable performance improvement has been demonstrated in both cross validation and case studies about colorectal cancer and lung cancer. Potential lncRNA-disease associations for all the disease investigated in this article have been publicly released for further biological experiment confirmation. In our opinion, LNCSIM has potential value for lncRNA-related interactions prediction and lncRNA biomarker detection for human disease diagnosis, treatment, prognosis and prevention.

There are at least three limitations in the method design of LNCSIM. Firstly, considering the fact that lncRNA functional similarity was calculated by integrating lncRNA-disease association data and the disease DAG, LNCSIM may cause bias to lncRNAs with more associated diseases. Therefore, the performance of LNCSIM would be further improved when more known experimentally verified disease-lncRNA associations can be obtained. Secondly, semantic contribution decay factor appear in the current model and how to select this parameter is not still solved well. Finally, lncRNA functional similarity calculation could be improved greatly by integrating more reliable types of biological datasets, such as lncRNA-related various interactions, lncRNA sequence, disease phenotype information.

## Methods

### LncRNA-disease associations

Considering accumulating biological experiments have produced hundreds of lncRNA–disease associations, we manually collected experimentally reported disease-lncRNA associations and constructed the first publicly available lncRNA–disease association database, LncRNADisease (http://cmbi.bjmu.edu.cn/lncrnadisease) in the previous work^11^, which aims to provide a comprehensive resource of experimentally confirmed lncRNA–disease associations and lay the data fundament for lncRNA-related predictive research. The lncRNA-disease association dataset was downloaded from the LncRNADisease database in October, 2012. In LncRNADisease database, the same disease-lncRNA association based on the different experimental literature evidences has been considered to be different associations. Therefore, 486 associations have been recorded in this database. We got rid of those duplicate associations based on different evidences for the same lncRNA-disease pair. As a result, 293 distinct high-quality experimentally verified lncRNA–disease associations have been obtained, including 118 lncRNAs and 167 diseases (see Supplementary Table 10).

To further analysis and validate the results of LNCSIM, we downloaded human lncRNA-disease associations in the MNDR^120^ in March, 2015. Similar to the operations for the dataset from LncRNADisease database, we got rid of those duplicate records based on different experimental literature evidences for the same lncRNA-disease associations. As a result, we obtained 471 high-quality experimentally verified human disease-lncRNA associations, including 127 diseases and 241 lncRNAs (see Supplementary Table 11).

### Disease MeSH descriptors and directed acyclic graph

MeSH descriptors of various diseases were downloaded from the National Library of Medicine (http://www.nlm.nih.gov/), which provided a strict system for disease classification for the research of the relationship among various diseases^121^. MeSH descriptors included 16 categories: Category A for anatomic terms, Category B for organisms, Category C for diseases, Category D for drugs and chemicals and so on. The MeSH descriptor of Category C for each disease was used in this paper. Furthermore, directed acyclic graph (DAG) ws constructed to demonstrate the relationship among various diseases, where the nodes represents disease MeSH descriptors and all the MeSH descriptors in the DAG are connected by a direct edge from a more general term (parent node) to a more specific term (child node) (See Fig. 1). Each MeSH descriptor has one or more tree numbers to numerically define its location in the DAG. The tree numbers of a child node are defined as the codes of its parent nodes appended by the child’s information. For the disease *A*, DAG is denoted as *DAG(A)* = *(D(A),E(A))*, where *D(A)* includes the nodes represent disease itself and its ancestor diseases and *E(A)* consisting of corresponding direct edges from a parent node to a child node represents the relationship between these two nodes (See Fig. 1).

Since the disease names in the LncRNADisease database and MNDR weren’t named based on MeSH descriptors, we mapped the diseases in these two disease-lncRNA association datasets into their MeSH descriptors. After getting rid of some diseases without any Mesh descriptors or tree numbers from these two disease-lncRNA association datasets and merging some diseases with the same Mesh descriptors, 254 and 260 distinct lncRNA-disease associations were obtained in LncRNADisease database and MNDR, respectively (see Supplementary Table 12 and 13).

### Disease semantic similarity model 1

As mentioned, functional similarity between two lncRNAs is calculated based on the similarity of diseases associated with these two lncRNAs. Therefore, we developed two models to calculate disease semantic similarity based on disease DAGs (See Fig. 1).

Firstly, we calculated the disease similarity in the same way as described in the literature^122^. Disease can be described as a DAG. We defined the contribution of disease term t in DAG(*A*) to the semantic value of disease *A* as follows:


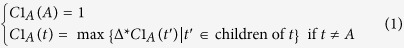


where 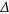 is the semantic contribution decay factor, which shows the contributions of other ancestor diseases to the semantic value of disease *A* decrease with the increase of the distance between this disease and disease *A*. In the DAG of disease *A*, disease *A* is located in the 0th layer, therefore it is the most specific disease term and its contribution to semantic value of disease *A* is defined as 1. Disease located in the 1st layer is considered to be a more general disease, so its contribution is multiplied by the semantic contribution decay factor. Based on above formula, the semantic contribution of diseases in different layers to semantic value of disease *A* are differentiated.

Therefore, summing all the contributions from ancestor diseases and disease *A* itself, the semantic value of disease *A* is defined as follows:


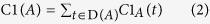


Furthermore, the semantic similarity between two diseases *A* and *B* can be defined based on the nodes shared by the two disease DAGs.


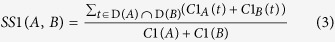


where *SS1* is the disease semantic similarity matrix. The entity *SS1(i,j)* in row *i* column *j* is the disease semantic similarity between disease *i* and *j* based on disease semantic similarity model 1.

### Disease semantic similarity model 2

Furthermore, the disease similarity was calculated in the same way as described in the literature^123^. According to disease semantic similarity model 1 defined above, the disease terms in the same layer of DAG(*A*) have the same contribution to the semantic value of disease *A*. However, different disease terms in the same layer of DAG(*A*) may appear in the different numbers of disease DAGs. For example, two diseases appear in the same layer of DAG(*A*) and the first disease appears in less disease DAGs than the second disease. Obviously, we can conclude that the first disease is more specific than the second disease. Therefore, it is less accurate to assign the same contribution value to these two diseases according to the above consideration. The contribution of the first disease to the semantic value of disease *A* should be higher than the second.

In conclusion, a more specific disease should have a greater contribution to the semantic value of disease *A*. Here, the contribution of disease term t in DAG(*A*) to the semantic value of disease *A* was defined as follows:


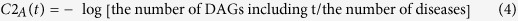


We defined the semantic similarity between disease *A* and *B* by summing all the contributions from ancestor diseases and disease *A* itself to define the semantic value of disease *A* in the similar way as model 1 and paying attention to the nodes shared by the two disease DAGs.


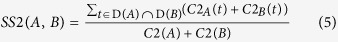


where *SS2* is the disease semantic similarity matrix calculated based on model 2, *C2(A)* and *C2(B)* is the semantic value of disease *A* and *B*, respectively. The entity *SS2(i,j)* in row *i* column *j* is the disease semantic similarity between disease *i* and *j* based on disease semantic similarity model 2.

### LNCSIM

Here, we developed the model of LNCSIM to quantitatively calculate lncRNA functional similarity by measuring the semantic similarity of their associated two groups of diseases (See Fig. 1). Taking the similarity calculation between lncRNA *u* and *v* as an example, we firstly defined *D(u)* and *D(v)* as the disease groups associated with lncRNA *u* and *v*, respectively. We calculated the similarity between *D(u)* and *D(v)* as the functional similarity between lncRNA *u* and *v*. To calculate the similarity between *D(u)* and *D(v)*, the similarity between one of diseases associate with one lncRNA and the group of diseases associated with the other lncRNA should be defined. The similarity between one of diseases associated with lncRNA *u*, such as D1, and the group of diseases associated with lncRNA *v* was calculated as follows:


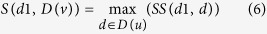


Finally, the functional similarity of lncRNA *u* and *v* was defined.


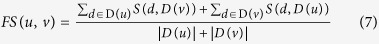


where *FS* is the lncRNA functional similarity matrix and the entity *FS(i,j)* in row *i* column *j* is the functional similarity between lncRNA *i* and *j*.

### Performance evaluation

LOOCV was implemented to compare the performance of LRLSLDA, LRLSLDA-LNCSIM1, and LRLSLDA-LNCSIM2. Each known disease-lncRNA association was used as test sample in turn and how well this association was ranked relative to the candidate disease-lncRNA pair was observed. In this way, all other known experimentally confirmed disease-lncRNA associations and all the disease-lncRNA pairs without confirmed associations were considered as training samples and candidate disease-lncRNA pair, respectively. Receiver operating characteristics (ROC) curve and Area under the curve (AUC) was used to implement performance evaluation. ROC curve plots true-positive rate (TPR, sensitivity) versus false-positive rate (FPR, 1-specificity) at different rank cutoffs. When the rank cutoffs of successful prediction were varied, corresponding TPR and FPR can be obtained. Here, sensitivity represents the percentage of the test samples obtaining the ranking higher than a given rank cutoff; Specificity represents the percentage of samples obtaining the ranking lower than this given rank cutoff. In this way, ROC was drawn and Area under the curve (AUC) was calculated. AUC = 1 indicates perfect performance and AUC = 0.5 indicates random performance.

## Additional Information

**How to cite this article**: Chen, X. *et al.* Constructing lncRNA functional similarity network based on lncRNA-disease associations and disease semantic similarity. *Sci. Rep.* **5**, 11338; doi: 10.1038/srep11338 (2015).

## Supplementary Material

Supplementary Information

Supplementary Table 1

Supplementary Table 2

Supplementary Table 3

Supplementary Table 4

Supplementary Table 5

Supplementary Table 6

Supplementary Table 7

Supplementary Table 8

Supplementary Table 9

Supplementary Table 10

Supplementary Table 11

Supplementary Table 12

Supplementary Table 13

## Figures and Tables

**Table 1:** As a global ranking method, LRLSLDA-LNCSIM1 was applied to simultaneously rank all the candidate lncRNA-disease associations.

**Disease**	**lncRNA**	**Evidence (PMID)**
Down’s syndrome	DGCR5	Unconfirmed
heroin abuse	MEG3	21128942
lung adenocarcinoma	H19	16707459
colorectal cancer	CRNDE	22393467
velocardiofacial syndrome	NRON	Unconfirmed
colorectal neoplasia	HOTAIR	24531795
lung adenocarcinoma	MEG3	Paper without PMID^124^
lung adenocarcinoma	BCYRN1	9422992
colorectal neoplasia	MALAT1	21503572
colorectal neoplasia	KCNQ1OT1	23660942
heroin addiction	MIAT	21128942
brain ischemia	B2 SINE RNA	15016078
liver injury	IFNG-AS1	Unconfirmed
cervix cancer	H19	8570220
breast cancer	MALAT1	24499465

The top 15 potential associations and the confirmation for their associations by experimental literature were listed here.
