# Supplementary material for: Constructing lncRNA functional similarity network based on lncRNA-disease associations and disease semantic similarity
Source: Sci Rep. 2015 Jun 10;5:11338. doi: 10.1038/srep11338 (PMC4462156; doi:10.1038/srep11338)
Supplement: Supplementary Information [file srep11338-s1.pdf]

Constructing lncRNA functional similarity network based on lncRNA-disease associations and disease semantic similarity

Xing Chen[1, 2,*,#], Chenggang Clarence Yan[3,#], Cai Luo[3],

Wen Ji[4], Yongdong Zhang[5], Qionghai Dai[3]

[1]National Center for Mathematics and Interdisciplinary Sciences,

Chinese Academy of Sciences, Beijing, 100190, China

[2]Academy of Mathematics and Systems Science,

Chinese Academy of Sciences, Beijing, 100190, China

[3]Department of Automation, Tsinghua University, Beijing, 100084, China

[4]Institute of Computing Technology,

Chinese Academy of Sciences, Beijing, 100190, China

[5] Key Lab of Intelligent Information Processing of Chinese Academy of Sciences, Institute of Computing Technology, Chinese Academy of Sciences, Beijing, 100190, China

*Corresponding authors

#The authors wish it to be known that, in their opinion, the first two authors should be regarded as joint First Authors.

Email: xingchen@amss.ac.cn

Supplementary Information

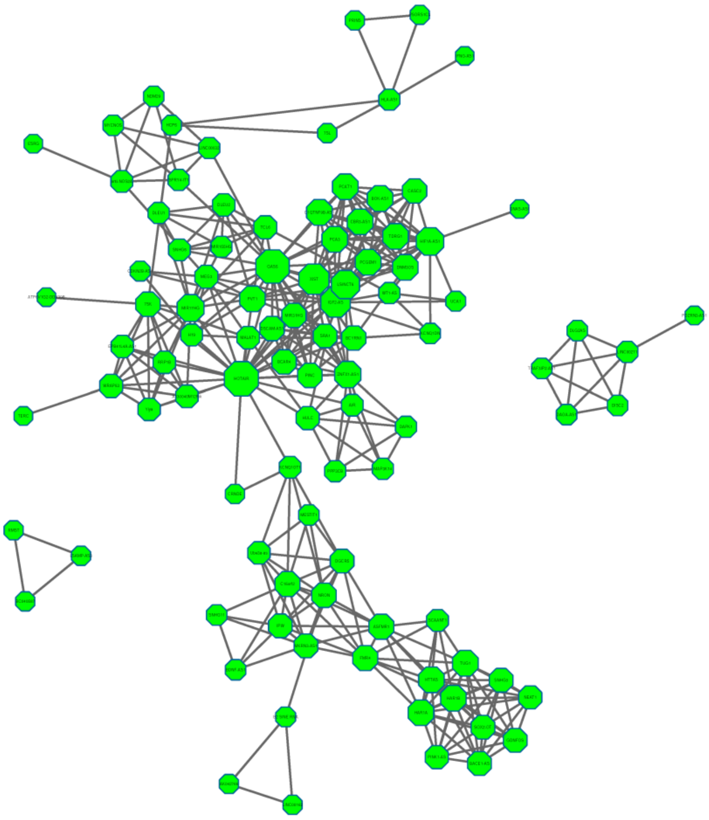

Supplementary Figure 1. lncRNA functional network was constructed by the model of LNCSIM based on disease semantic similarity model 2, where each node represents one lncRNA and the links was connected if lncRNA pair has a functional similarity equal to or greater than the similarity cutoff (here the cutoff is 0.3 considering the fact that known lncRNA-disease associations is seriously incomplete currently). The size of a node is proportional to the degree of the node. The network is visualized by cytoscape (http://cytoscape.github.io/).

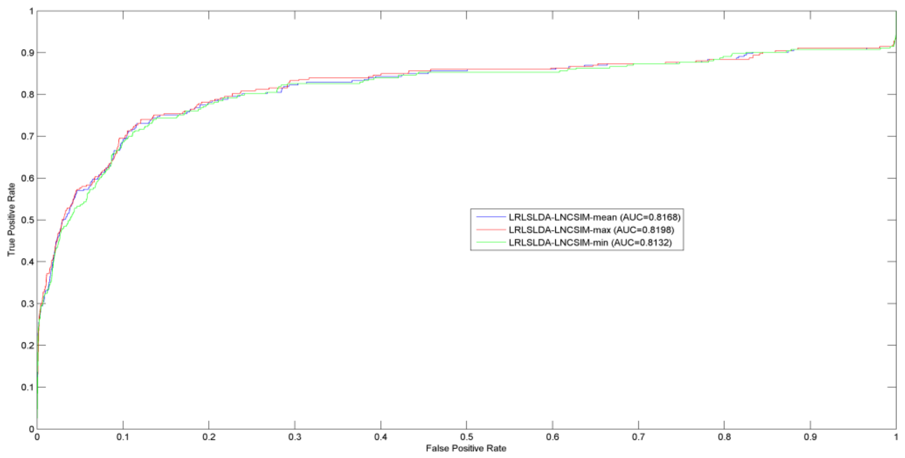

Supplementary Figure 2. Comparison between LRLSLDA-LNCSIM-mean, LRLSLDA-LNCSIM-max, and LRLSLDA-LNCSIM-min in terms of ROC curve and AUC based on LOOCV. As a result, LRLSLDA-LNCSIM-mean, LRLSLDA-LNCSIM-max, and LRLSLDA-LNCSIM-min achieved AUCs of 0.8168, 0.8199, and 0.8132, respectively (see Supplementary Figure 2). No significant performance differences from LRLSLDA-LNCSIM1 and LRLSLDA-LNCSIM2 could be observed, which indicated the similarity results based on LNCSIM1 and LNCSIM2 are not complementary.

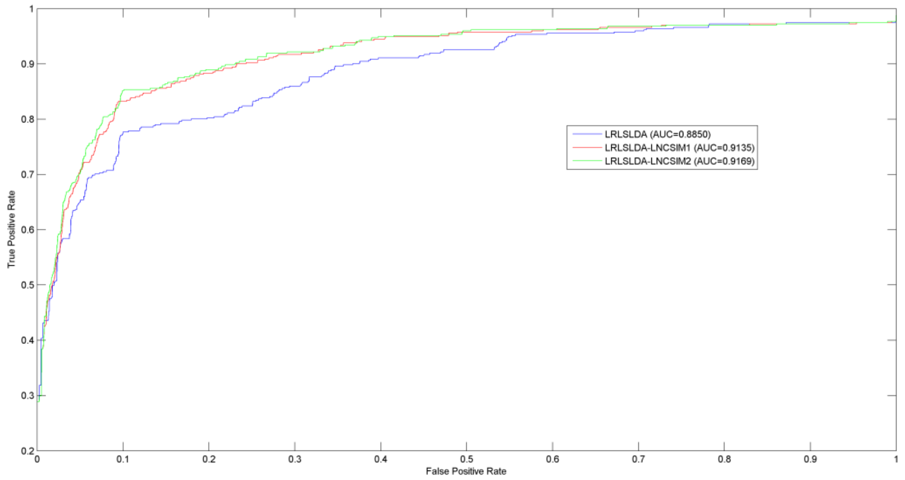

Supplementary Figure 3. Comparison between LRLSLDA, LRLSLDA-LNCSIM1, and LRLSLDA-LNCSIM2 based on LOOCV implemented on the dataset from MNDR. As a result, predictive accuracy has been improved by the operation of introducing new disease similarity and lncRNA functional similarity calculated from LNCSIM.

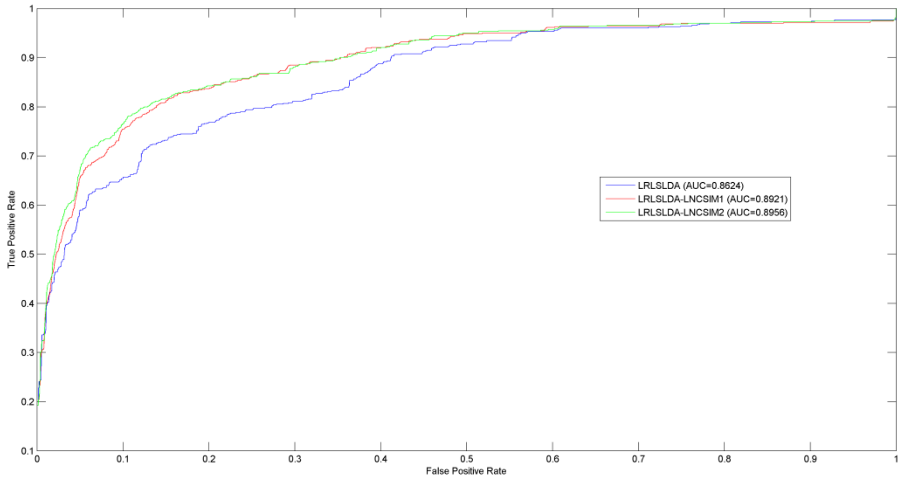

Supplementary Figure 4. Comparison between LRLSLDA, LRLSLDA-LNCSIM1, and LRLSLDA-LNCSIM2 based on LOOCV implemented on the integrated dataset. As a result, predictive accuracy has been improved by the operation of introducing new disease similarity and lncRNA functional similarity calculated from LNCSIM.

Supplementary Table 1. Pairwise functional similarity among 104 lncRNAs investigated in the LncRNADisease database calculated by the model of LNCSIM based on disease semantic similarity model 1.

Supplementary Table 2. Pairwise functional similarity among 104 lncRNAs investigated in the LncRNADisease database calculated by the model of LNCSIM based on disease semantic similarity model 2.

Supplementary Table 3. As a global ranking method, LRLSLDA-LNCSIM2 was applied to simultaneously rank all the candidate lncRNA-disease associations. The top 15 potential associations and the confirmation for their associations by experimental literature were listed here.

Supplementary Table 4. Potential human disease-lncRNA association list for each disease predicted by LRLSLDA-LNCSIM1 were publicly released to benefit the biological experimental validation.

Supplementary Table 5. Potential human disease-lncRNA association list for each disease predicted by LRLSLDA-LNCSIM2 were publicly released to benefit the biological experimental validation.

Supplementary Table 6. Pairwise functional similarity among 95 lncRNAs investigated in the MNDR calculated by the model of LNCSIM based on disease semantic similarity model 1.

Supplementary Table 7. Pairwise functional similarity among 95 lncRNAs investigated in the MNDR calculated by the model of LNCSIM based on disease semantic similarity model 2.

Supplementary Table 8. Pairwise functional similarity among 169 lncRNAs investigated in the integrated dataset calculated by the model of LNCSIM based on disease semantic similarity model 1.

Supplementary Table 9. Pairwise functional similarity among 169 lncRNAs investigated in the integrated dataset calculated by the model of LNCSIM based on disease semantic similarity model 2.

Supplementary Table 10. The lncRNA-disease association dataset was downloaded from the LncRNADisease database in October, 2012. After getting rid of duplicate associations, this dataset consists of 293 distinct high-quality experimentally verified lncRNA − disease associations, including 118 lncRNAs and 167 diseases.

Supplementary Table 11. The lncRNA-disease association dataset was downloaded from MNDR in March, 2015. After getting rid of duplicate associations, this dataset consists of 471 distinct high-quality experimentally verified lncRNA − disease associations, including 241 lncRNAs and 127 diseases.

Supplementary Table 12. After getting rid of some diseases without any Mesh descriptors or tree numbers from disease-lncRNA association dataset in the LncRNADisease database and merging some diseases with the same Mesh descriptors, 254 distinct lncRNA-disease associations were obtained, including 104 lncRNAs and 126 diseases.

Supplementary Table 13. After getting rid of some diseases without any Mesh descriptors or tree numbers from disease-lncRNA association dataset in the MNDR and merging some diseases with the same Mesh descriptors, 260 distinct lncRNA-disease associations were obtained, including 95 lncRNAs and 81 diseases.
